# Relationship of paroxysmal nocturnal hemoglobinuria (PNH) granulocyte clone size to disease burden and risk of major vascular events in untreated patients: results from the International PNH Registry

**DOI:** 10.1007/s00277-023-05269-4

**Published:** 2023-05-18

**Authors:** David Dingli, Jaroslaw P. Maciejewski, Loree Larratt, Ronald S. Go, Britta Höchsmann, Ke Zu, Philippe Gustovic, Alexander D. Kulagin

**Affiliations:** 1 Division of Hematology, Mayo Clinic, 200 First Street SW, Rochester, MN 55905 USA; 2 Department of Translational Hematology and Oncology Research, Taussig Cancer Institute, Cleveland Clinic, Cleveland, OH USA; 3 Division of Hematology, University of Alberta, Edmonton, AB Canada; 4 Institute of Transfusion Medicine, University of Ulm, and Institute for Clinical Transfusion Medicine and Immunogenetics, German Red Cross Blood Transfusion Service Baden-Württemberg-Hessen and University Hospital Ulm, Ulm, Germany; 5 Alexion, AstraZeneca Rare Disease, Boston, MA USA; 6 Alexion, AstraZeneca Rare Disease, Zürich, Switzerland; 7 RM Gorbacheva Research Institute, Pavlov University, Saint Petersburg, Russia

**Keywords:** Paroxysmal nocturnal hemoglobinuria, Cohort studies, Disease progression, GPI-deficient granulocytes, Risk factors, Thromboembolism

## Abstract

Paroxysmal nocturnal hemoglobinuria (PNH) is caused by acquired gene mutations resulting in deficiency of glycosylphosphatidylinositol (GPI)–anchored complement regulatory proteins on the surface of blood cells, leading to terminal complement–mediated intravascular hemolysis and increased risk of major adverse vascular events (MAVEs). Using data from the International PNH Registry, this study investigated the relationship between the proportion of GPI-deficient granulocytes at PNH onset and (1) the risk for MAVEs (including thrombotic events [TEs]) and (2) the following parameters at last follow-up: high disease activity (HDA); lactate dehydrogenase (LDH) ratio; fatigue; abdominal pain; and rates of overall MAVEs and TEs. A total of 2813 patients untreated at enrollment were included and stratified by clone size at PNH disease onset (baseline). At last follow-up, higher proportion of GPI-deficient granulocytes (≤ 5% vs. > 30% clone size) at baseline was associated with significantly increased HDA incidence (14% vs. 77%), mean LDH ratio (1.3 vs. 4.7 × upper limit of normal), and rates of MAVEs 1.5 vs. 2.9 per 100 person-years) and TEs (0.9 vs. 2.0 per 100 person-years). Fatigue was evident in 71 to 76% of patients regardless of clone size. Abdominal pain was more frequently reported with clone size > 30%. A larger clone size at baseline appears to indicate a greater disease burden and risk of TEs and MAVEs and may inform decision making among physicians managing PNH patients at risk of experiencing TEs or other MAVEs. ClinicalTrials.gov ID: NCT01374360.

**Supplementary Information:**

The online version contains supplementary material available at 10.1007/s00277-023-05269-4.

## Introduction

Paroxysmal nocturnal hemoglobinuria (PNH) is an acquired clonal hematopoietic stem cell disease characterized by terminal complement–mediated intravascular hemolysis (IVH) and thrombophilia [1]. The prevalence of PNH is estimated to be approximately 38 per million individuals [2], with an incidence of 0.08 to 0.57 per 100,000 person-years [2–4]. PNH is caused by somatic mutations of the phosphatidylinositol-glycan (*PIG-A*) gene that result in deficiency of the glycophosphatidylinositol (GPI)–anchored complement regulatory proteins CD55 and CD59 on the surface of blood cells, including red and white blood cells and platelets [5–7]. Loss of these complement regulators results in IVH (elevated lactate dehydrogenase [LDH]); renal impairment; pulmonary hypertension; anemia; fatigue; abdominal pain; and an increased risk of major adverse vascular events (MAVEs), including thrombotic events (TEs) [1, 8]. TE is the leading cause of mortality among individuals with PNH, accounting for approximately 40 to 67% of deaths with a known cause in this population [5, 9, 10].

Treatment decisions for PNH are informed by several clinical parameters, including elevated LDH; presence of signs and symptoms such as anemia, abdominal pain, chest pain, and renal dysfunction; and for some physicians, by the proportion of GPI-deficient granulocytes [11]. Currently, the longitudinal relationship between GPI-deficient clone size and risk of MAVEs, including TE, over time remains unclear. Although patients with a proportion of GPI-deficient granulocytes > 50% experienced more debilitating symptoms in prior studies, the relationship between clone size and clinical outcomes is not linear, and the risk of TE exists even in patients with a clone size of ≤ 10%, suggesting a need for continued therapy [12, 13]. Previous studies assessing TE and other MAVEs have shown a positive correlation between clone size and MAVE risk; however, most of these studies have been limited by small sample sizes or were cross-sectional analyses [12–14].

The ongoing International PNH Registry (NCT01374360) is the largest prospective, global, observational study of patients with PNH to date and has continuously collected patient data since its initiation in 2007 [15]. The Registry includes data for patients with a confirmed diagnosis of PNH and/or a detectable clone size down to 0.01%, allowing for evaluation of the relationship between clone size and clinical outcomes. The objective of this analysis was to examine the relationship between PNH clone size at disease onset and MAVE/TE rates as well as the frequency of high disease activity (HDA) and PNH-related symptoms (e.g., fatigue) in patients who were enrolled in the International PNH Registry and were not treated with a complement inhibitor. A greater understanding of the prognostic value of clone size may better inform clinical decision making and improve patient outcomes.

## Methods

### Patient population

The study population included patients enrolled in the International PNH Registry with a valid patient ID; known date of birth, sex, enrollment date, and treatment status; PNH clone size (i.e., proportion of GPI-deficient granulocytes ≥ 0.01% determined by flow cytometry) reported at or before Registry enrollment; and ≥ 12 months between baseline and last follow-up. Patients were included in the study if they had not been treated with eculizumab at enrollment. Baseline was defined as PNH onset (i.e., disease start date) at the earliest reported GPI-deficient clone, date of PNH diagnosis, or PNH symptom. Last follow-up was defined as last Registry follow-up or as the point before eculizumab initiation. The patient population was stratified into 5 cohorts based on first reported PNH clone size on or before enrollment: ≤ 5%, > 5 to ≤ 10%, > 10 to ≤ 30%, and > 30%. Clone size categories were determined arbitrarily.

### Assessments

Patient demographics and medical history variables collected at baseline included sex, race, age at baseline, history of bone marrow failure (BMF), history of MAVEs, and history of concomitant medications. History of clinical events such as MAVEs was defined as having a record and/or date of the event recorded before baseline. History of concomitant medications was defined as having an assessment date or any start or end date at or before or at baseline. BMF included aplastic anemia (AA), myelodysplastic syndromes, acute myelogenous leukemia, myelofibrosis, and other bone marrow pathologies. MAVEs included TE MAVEs (i.e., thrombophlebitis/deep vein thrombosis, renal vein thrombosis, renal arterial thrombosis, mesenteric/visceral vein thrombosis, mesenteric/visceral arterial thrombosis, hepatic/portal vein thrombosis, dermal thrombosis, acute peripheral vascular disease occlusion, cerebral arterial occlusion/cerebrovascular accident, cerebral venous occlusion, and pulmonary embolus), as well as nontraumatic or nondiabetic amputation, myocardial infarction, transient ischemic attack, unstable angina, nontraumatic or nondiabetic gangrene, and other MAVEs. MAVE rates were calculated in the time between baseline and last follow-up. Concomitant medications evaluated included immunosuppressive therapy with cyclosporine or anti-thymocyte globulin (ATG), immunosuppressive therapy with corticosteroids, any oral prophylactic antibiotics, pain medication (any, opioid, or nonopioid), and anticoagulation therapy with heparin or warfarin.

Outcomes assessed included HDA, LDH ratio, symptoms of fatigue and abdominal pain, estimated glomerular filtration rate (eGFR), red blood cell (RBC) transfusion needs, and MAVEs (from baseline through last follow-up). HDA was defined as LDH ratio ≥ 1.5 × upper limit of normal (ULN) within 6 months before and including last untreated follow-up date and ≥ 1 of the following: history of MAVEs (including TE MAVEs), anemia (defined as hemoglobin < 10 g/dL), or history of physician-documented symptoms of abdominal pain, dyspnea, dysphagia, fatigue, hemoglobinuria, or erectile dysfunction at any time before and including last untreated follow-up date. Duration of follow-up (from baseline to last follow-up) and disposition were also evaluated.

### Statistical analyses

Data, stratified by clone size cohort at baseline, were summarized using mean ± SD and median (Q1, Q3) for continuous variables and frequency and percentage for categorical variables. MAVE rates were also estimated for each cohort per 100 person-years and presented with 95% CI. In an adjusted analysis of MAVE rates, estimates included the LDH ratio and history of BMF at baseline as covariates. Person-years were calculated as the time, in years, between baseline and the last follow-up date for all patients regardless of whether they experienced an event. The event rate was calculated using Poisson regression.

## Results

### Demographics, baseline characteristics, and disposition of patients with PNH

Of 5302 patients in the Registry as of data lock on July 8, 2019, data from 2813 patients met the inclusion criteria. Patients were grouped into the following cohorts based on clone size at baseline: ≤ 5%, *n* = 1006; > 5 to ≤ 10%, *n* = 221; > 10 to ≤ 30%, *n* = 443; and > 30%, *n* = 1143 (Table 1). These cohort sizes translate to 35.8%, 7.9%, 15.7%, and 40.6% of the analysis population, respectively. Patient demographics and characteristics were generally balanced among the clone size cohorts. In all cohorts, most patients were white (72.2–83.8%) and a slight majority were female (52.3–57.3%). Median (Q1, Q3) age at PNH onset in all cohorts ranged from 33 (23.5, 46.9) to 44 (26.6, 61.4) years, with slightly younger patients in the > 5 to ≤ 10%, and > 30% clone size cohorts. There were slight differences in the proportion of Asian patients among clone size cohorts, with larger clone size at baseline associated with an increasing proportion of Asian patients. The percentage of patients with history of BMF at baseline was higher in the lower clone size cohorts, ranging from 54.2% (clone size ≤ 5%) to 31.3% (clone size > 30%). Immunosuppressant use was generally lower with increasing clone size; this trend was not observed with other concomitant medications (i.e., anticoagulation therapy), which were used by ≤ 3% of patients in any cohort (Supplementary Fig. 1). Immunosuppressive therapies were the most frequent concomitant medications at baseline in all clone size cohorts, with a greater percentage of patients in each cohort receiving cyclosporine or ATG vs. corticosteroids.

**Table 1:** Baseline^a^ demographics and characteristics of patients with PNH^b^

	≤ 5% (*n* = 1006)	> 5 to ≤ 10% (*n* = 221)	> 10 to ≤ 30% (*n* = 443)	> 30% (*n* = 1143)
Sex, *n* (%)				
Female	526 (52.3)	121 (54.8)	254 (57.3)	602 (52.7)
Male	480 (47.7)	100 (45.2)	189 (42.7)	541 (47.3)
Race, *n* (%)				
White or Caucasian descent	841 (83.8)	176 (80.0)	337 (76.4)	823 (72.2)
Asian	125 (12.5)	34 (15.5)	76 (17.2)	276 (24.2)
Black or African descent	23 (2.3)	5 (2.3)	14 (3.2)	25 (2.2)
Native or Aboriginal	0	2 (0.9)	3 (0.7)	2 (0.2)
Unlisted or multiple races	15 (1.5)	3 (1.4)	11 (2.5)	14 (1.2)
Age at PNH onset, y, median (Q1, Q3)	44 (26.6, 61.4)	35 (22.7, 53.6)	38 (25.0, 55.6)	33 (23.5, 46.9)
History of BMF,^b^ n (%)	539 (54.2)	113 (51.8)	192 (43.8)	352 (31.3)
History of TE, n (%)	33 (3.4)	6 (2.8)	8 (1.8)	46 (4.1)

*BMF*, Bone marrow failure; *GPI*, glycophosphatidylinositol; *MAVE*, major adverse vascular event; *PNH*, paroxysmal nocturnal hemoglobinuria; *TE*, thrombotic event

^a^Baseline was defined as PNH onset (i.e., disease start date) at the earliest reported GPI-deficient clone, date of PNH diagnosis, or PNH symptom

^b^Includes aplastic or hypoplastic anemia, acute myelogenous leukemia, myelodysplastic syndrome, myelofibrosis, and other bone marrow pathology

Larger PNH clone size at baseline was associated with a greater duration from baseline to last untreated follow-up, ranging from 6 ± 6.7 years in patients with clone size ≤ 5 to 9.7 ± 9.2 years in patients with clone size > 30% (Supplementary Table 1). The most common reasons for last untreated follow-up visit varied across cohorts: last contact in the Registry or withdrawal from the Registry (not owing to bone marrow transplant or death) were the most common reasons in smaller clone size cohorts (last contact: 48.1–53.0%, withdrawal: 23.1–34.0%), whereas eculizumab initiation was the most common reason in the largest clone size cohort (41.3%). Death and bone marrow transplant were uncommon in all cohorts.

### Clinical parameters at last follow-up

**Fig. 1:**
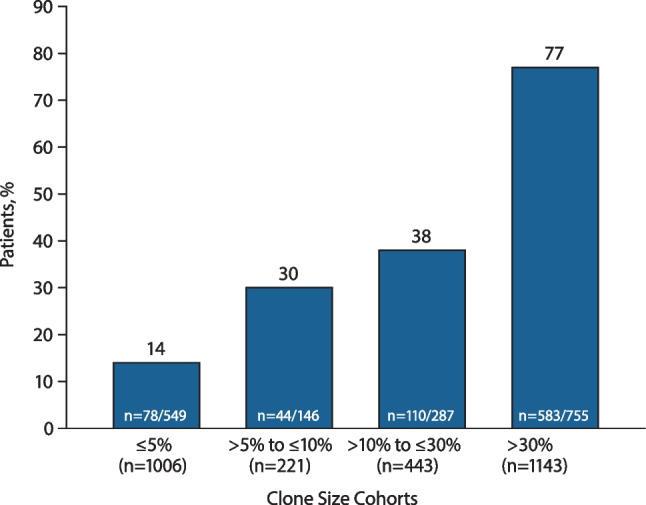
Patients with high disease activity at last follow-up stratified by clone size at baseline. High disease activity was defined as LDH ratio ≥ 1.5 × ULN within 6 months before and including baseline *and* ≥ 1 of the following: history of MAVEs (including TEs) at any time before baseline, anemia (defined as hemoglobin < 10 g/dL) at any time before and including baseline, or history of physician-documented symptoms of abdominal pain, dyspnea, dysphagia, fatigue, hemoglobinuria, or erectile dysfunction at any time before baseline. Baseline was defined as PNH onset (i.e., disease start date) at the earliest reported GPI-deficient clone, date of PNH diagnosis, or PNH symptom. N indicates total number of patients at risk. Data within bars reflect number of patients with high disease activity / total number of patients with nonmissing data. GPI, glycophosphatidylinositol; LDH, lactate dehydrogenase; MAVE, major adverse vascular event; PNH, paroxysmal nocturnal hemoglobinuria; TE, thrombotic event; ULN, upper limit of normal

**Fig. 2:**
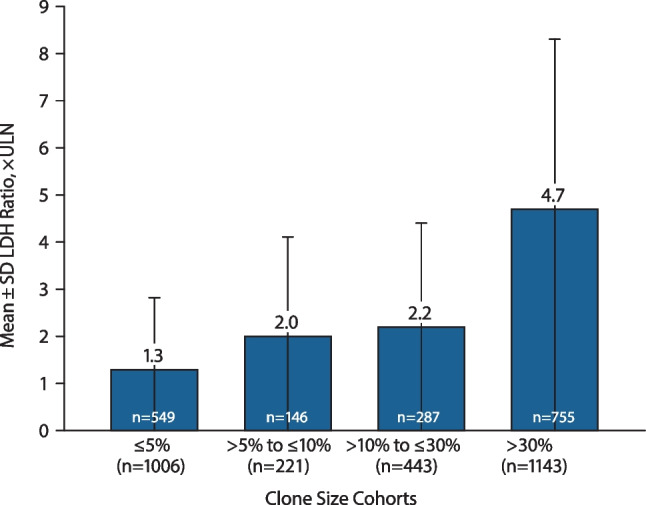
LDH ratio × ULN at last follow-up stratified by clone size at baseline. Baseline was defined as PNH onset (ie, disease start date) at the earliest reported GPI-deficient clone, date of PNH diagnosis, or PNH symptom. Data within bars reflects total number of patients with nonmissing data. GPI, glycophosphatidylinositol; LDH, lactate dehydrogenase; PNH, paroxysmal nocturnal hemoglobinuria; ULN, upper limit of normal

The percentage of patients with HDA at last follow-up increased across increasing baseline clone size cohorts (Fig. 1): 14% in the clone size ≤ 5% cohort and 77% in the clone size > 30% cohort. Mean ± SD LDH ratio × ULN at last follow-up was likewise greater among patients with larger baseline clone size (Fig. 2); LDH ratio at last follow-up was 1.3 × ULN in the clone size ≤ 5% cohort and 4.7 × ULN in the clone size > 30% cohort.

**Fig. 3:**
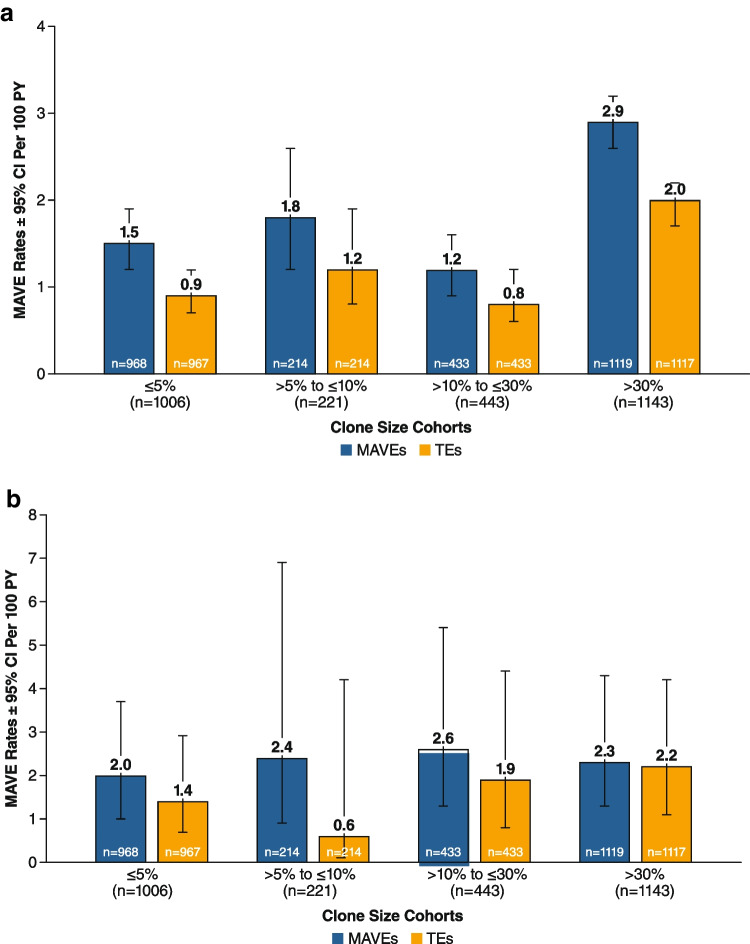
Rates of MAVEs from baseline to last follow-up stratified by clone size at baseline. **A** Unadjusted model. **B** adjusted model. Baseline was defined as PNH onset (i.e., disease start date) at the earliest reported GPI-deficient clone, date of PNH diagnosis, or PNH symptom. MAVEs included TE MAVEs. Data within bars reflect total number of patients with nonmissing data. BMF, bone marrow failure; GPI, glycophosphatidylinositol; LDH, lactate dehydrogenase; MAVE, major adverse vascular event; PNH, paroxysmal nocturnal hemoglobinuria; PY, person-years; TE, thrombotic event

Rates of MAVEs were calculated per 100 person-years; the person-years were 5846.6, 1568.2, 3446.4, and 10,895.6 for the clone size ≤ 5%, > 5 to ≤ 10%, > 10 to ≤ 30%, and > 30% cohorts, respectively. The most frequently reported MAVEs among the different cohorts (clone size ≤ 5%, > 5 to ≤ 10%, > 10 to ≤ 30%, and > 30%) included thrombophlebitis/deep vein thrombosis (49%, 32%, 48%, and 25%, respectively), pulmonary embolus (23%, 32%, 10%, and 11%), hepatic/portal vein thrombosis (6%, 11%, 10%, and 22%), cerebral arterial occlusion/cerebrovascular accident (4%, 16%, 17%, and 12%), and mesenteric/visceral vein thrombosis (4%, 0%, 3%, and 12%). Larger clone size at baseline was associated with higher rates of MAVEs (including TE MAVEs) through last follow-up visit (Fig. 3A, B). Rates of MAVEs were higher in patients with a clone size > 30% (2.9 per 100 person-years) than in patients with clone size ≤ 5% (1.5 per 100 person-years). A similar trend was observed for TE, which comprised the majority of MAVEs in all clone size cohorts; rates for TE MAVEs ranged from 0.9 per 100 person-years in the clone size ≤ 5% cohort to 2.0 per 100 person-years in the clone size > 30% cohort.

The majority of patients in this analysis had documented fatigue at the last follow-up visit regardless of clone size at baseline; the percentage of patients with fatigue ranged from 70.9 to 76.3% across cohorts (Supplementary Fig. 2). Approximately 32.4% of the patients had abdominal pain at the last follow-up visit, and there was a trend toward more patients with abdominal pain in the larger baseline clone size cohorts (40.1% of patients with clone size > 30% vs. 23.4 to 28.9% of patients with clone size ≤ 30%). Patients with baseline clone size ≤ 5% exhibited lower mean ± SD eGFR at last follow-up (81.2 ± 27.2 mL/min) compared with those with > 30% clone size (94.8 ± 27.8 mL/min; Supplementary Table 2); however, there was no clear association between clone size and eGFR across cohorts. Finally, there was no clear association between RBC transfusion needs and clone size at baseline (Supplementary Table 3).

## Discussion

This is the first study with a substantial sample size to examine the relationship between the proportion of GPI-deficient granulocytes at disease onset and MAVE rates (including TE MAVEs), as well as disease burden in untreated patients with PNH. Larger clone size was directly proportional to an increased proportion of patients with HDA at last follow-up and increased rates of overall MAVEs and TE MAVEs. Further, there was also a trend of higher LDH ratio as well as a greater proportion of patients with abdominal pain among those with larger baseline clone size. There was no clear relationship between clone size and RBC transfusion needs, eGFR, or fatigue. However, fatigue was reported in the majority of the population across all cohorts, which likely reflects the contribution of both intravascular hemolysis and anemia that may in part be caused by BMF, as well as lower eGFR that could be attributed, at least in part, to the use of cyclosporine.

Among the patient cohorts stratified by clone size at baseline, there were slight differences between clone size at baseline and the age of PNH onset, as well as between baseline clone size and race, specifically the proportion of Asian patients. Approximately one-third of patients had baseline clone size ≤ 5%, which may reflect earlier detection owing to improvements in disease awareness and increased emphasis on screening (particularly in patients with BMF syndromes or unexplained cytopenias) [1, 16, 17]. For example, patients with small clone size may not necessarily have many clinical symptoms related to PNH; however, these patients with a small clone would usually be diagnosed after being screened for PNH owing to BMF. Clone sizes at baseline and history of BMF were inversely proportional, with BMF approximately twice as common in the smallest clone size cohort (≤ 5%) compared with the largest clone size cohort (> 30%). This is not unexpected, as patients with no history of BMF and small clone sizes would not be screened owing to their lack of HDA. Because patients with AA are screened, small clone sizes are commonly detected in these patients; further, BMF may be masked among those with classical PNH in patients with very large clone size by the PNH clone itself, and in those with AA owing to response to immunosuppressive therapy and restoration of hematopoiesis [18]. Immunosuppressive therapy with cyclosporine or ATG was the most commonly reported concomitant medication used at baseline, and the greatest percentage of patients receiving these therapies was evident among those with the smallest clone size. Corticosteroid use was also very common and followed the same trend. Use of these therapies may reflect, in part, management of underlying AA, including prevention of serum sickness with ATG [19]. Moreover, patients with clone size ≤ 5% also exhibited lower mean eGFR at last follow-up compared with those with > 30% clone size. Nephrotoxicity has been associated with cyclosporine use.

At last follow-up, the percentage of patients per cohort with HDA as well as the LDH ratio was directly proportional to clone size at baseline. Although fatigue was universally prevalent in this study, abdominal pain occurred at a greater rate in patients with large clone size. These data indicate that clone size at PNH onset may be a prognostic longitudinal factor for disease burden, including both HDA and clinical symptoms. This is also in agreement with the observation that the most common reason for last follow-up in the clone size > 30% cohort was initiation of eculizumab treatment, which was less common in smaller clone size cohorts.

Use of anticoagulation therapy with heparin or warfarin at baseline was uncommon in the patients evaluated, with only 1% of patients with small clone size and 3% of patients with large clone size receiving these treatments despite the known risk of TE. Rates of both all-cause MAVEs and TE MAVEs were significantly increased with higher baseline clone size. MAVE rates were approximately twofold higher in the large clone size cohort compared with smaller clone size cohorts, almost exceeding 3 per 100 person-years between baseline and last follow-up; this was also the case for TE-specific MAVEs. These data confirm and extend previous studies demonstrating a relationship between clone size and MAVEs [10, 12, 14, 20] and suggest prognostic value of clone size in evaluating MAVE risk, which may inform treatment decisions regarding initiation or maintenance of therapy.

Some limitations of this analysis include the observational nature of the Registry and the fact that not every patient included had data available for every outcome assessed, leading to an inconsistent number of patients contributing data to each assessment. However, use of Registry data allowed evaluation of a large number of patients both overall and across individual cohorts in this rare disease, which increases confidence in our findings. Patient data were censored at the time of eculizumab treatment initiation, which may have implications on these findings as well as shorter follow-up in patients with HDA who were presumably treated with eculizumab. Additionally, patients with hemolysis and severe disease would most likely be treated with eculizumab before enrollment and thus were not included in this analysis.

In conclusion, this analysis of International PNH Registry data suggests a correlation between clone size and disease burden/risk of MAVEs, which may have prognostic value related to physician decision making in treating patients with PNH at risk of MAVEs.

## Supplementary Information

Below is the link to the electronic supplementary material.

Fig. S1 (PNG 117 kb)

High resolution image (EPS 1683 kb)

Fig. S2 (PNG 91 kb)

High resolution image (EPS 1589 kb)

Supplementary Table 1 (DOCX 20 kb)

Supplementary Table 2 (DOCX 20 kb)

Supplementary Table 3 (DOCX 20 kb)

## Data Availability

Alexion, AstraZeneca Rare Disease will consider requests for disclosure of clinical study participant-level data provided that participant privacy is assured through methods such as data de-identification, pseudonymization, or anonymization (as required by applicable law), and if such disclosure was included in the relevant study informed-consent form or similar documentation. Qualified academic investigators may request participant-level clinical data and supporting documents (statistical analysis plan and protocol) pertaining to Alexion-sponsored studies. Further details regarding data availability and instructions for requesting information are available in the Alexion Clinical Trials Disclosure and Transparency Policy at http://alexion.com/our-research/research-and-development. Link to data-request form: https://alexion.com/contact-alexion/medical-information.
