# Supplementary figures and images for: Relationship of paroxysmal nocturnal hemoglobinuria (PNH) granulocyte clone size to disease burden and risk of major vascular events in untreated patients: results from the International PNH Registry
Source: Ann Hematol. 2023 May 18;102(7):1637–44. doi: 10.1007/s00277-023-05269-4 (PMC10261189; doi:10.1007/s00277-023-05269-4)

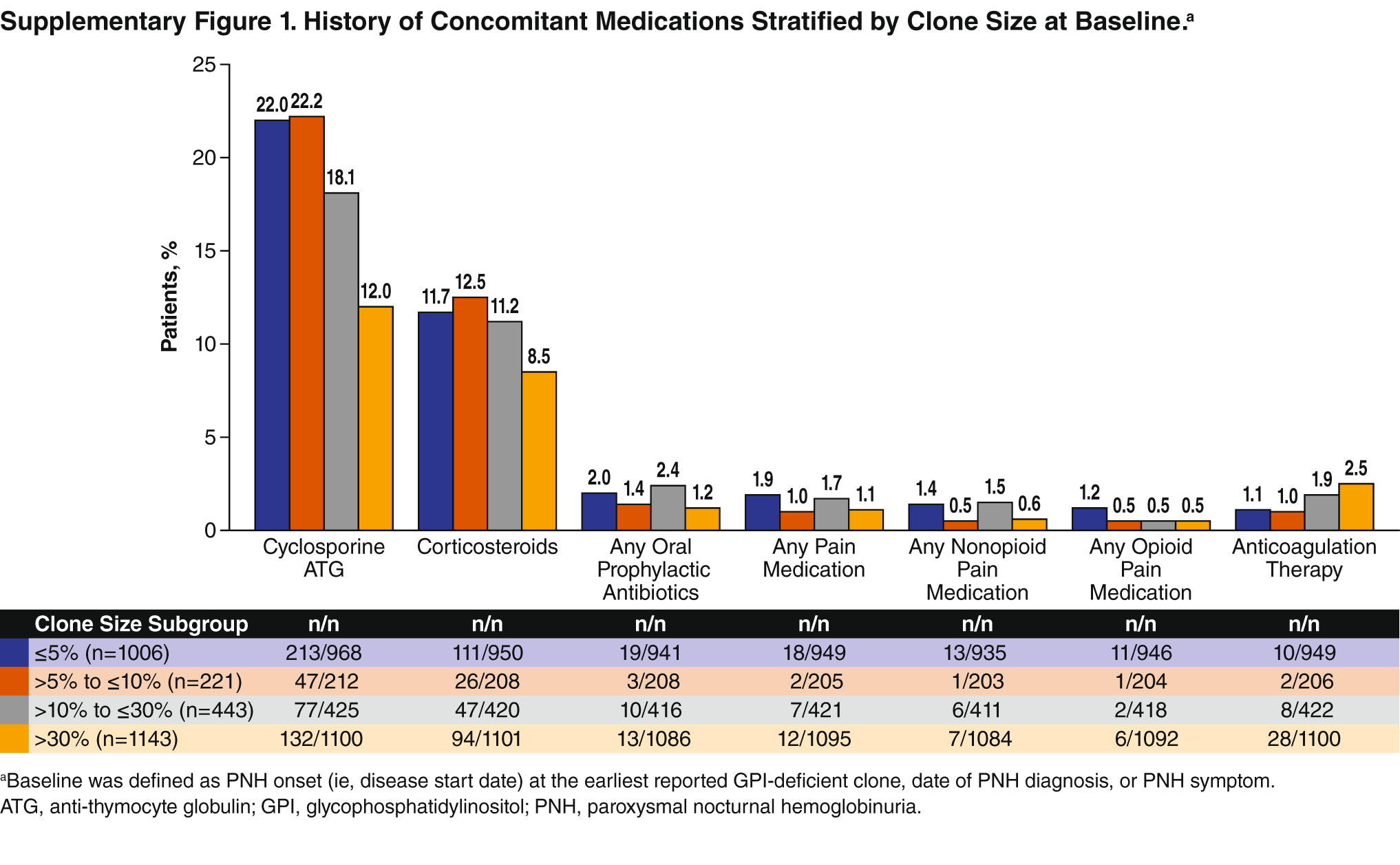

Supplement: Supplementary file 1 — (PNG 117 kb) [file 277_2023_5269_Fig4_ESM.png]

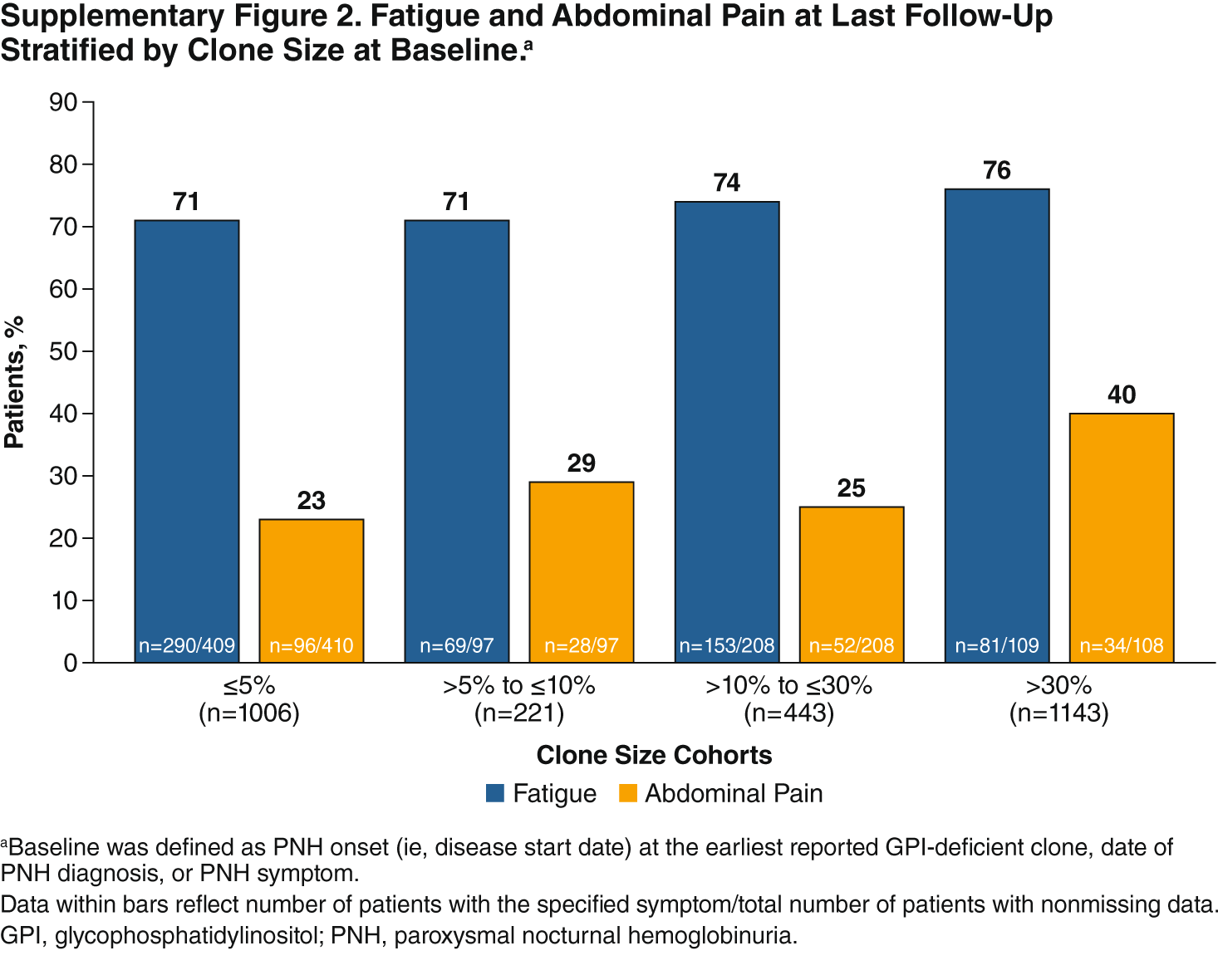

Supplement: Supplementary file 3 — (PNG 91 kb) [file 277_2023_5269_Fig5_ESM.png]
