# Supplementary material for: Relationship of paroxysmal nocturnal hemoglobinuria (PNH) granulocyte clone size to disease burden and risk of major vascular events in untreated patients: results from the International PNH Registry
Source: Ann Hematol. 2023 May 18;102(7):1637–44. doi: 10.1007/s00277-023-05269-4 (PMC10261189; doi:10.1007/s00277-023-05269-4)
Supplement: Supplementary file 5 — (DOCX 20 kb) [file 277_2023_5269_MOESM3_ESM.docx]

**Supplementary Table 1. Patient Duration of Follow-Up and Disposition Stratified by Clone Size at Baseline^a^**

|  | **≤5%**  **(n=1006)** | **>5% to ≤10%**  **(n=221)** | **>10% to ≤30%**  **(n=443)** | **>30%**  **(n=1143)** |
| --- | --- | --- | --- | --- |
| Baseline to last follow-up, y, mean ± SD | 6 (6.7) | 7 (7.1) | 8 (7.1) | 10 (9.2) |
| Reason for last follow-up, n (%) |  |  |  |  |
| Last contact in Registry | 533 (53.0) | 115 (52.0) | 213 (48.1) | 301 (26.3) |
| Withdrew from the Registry | 342 (34.0) | 51 (23.1) | 122 (27.5) | 288 (25.2) |
| Death | 77 (7.7) | 14 (6.3) | 24 (5.4) | 56 (4.9) |
| Eculizumab initiated | 29 (2.9) | 29 (13.1) | 73 (16.5) | 472 (41.3) |
| Bone marrow transplant | 25 (2.5) | 12 (5.4) | 11 (2.5) | 26 (2.3) |

GPI, glycophosphatidylinositol; PNH, paroxysmal nocturnal hemoglobinuria.

^a^Baseline was defined as PNH onset (ie, disease start date) at the earliest reported GPI-deficient clone, date of PNH diagnosis, or PNH symptom.
