# Supplementary material for: Relationship of paroxysmal nocturnal hemoglobinuria (PNH) granulocyte clone size to disease burden and risk of major vascular events in untreated patients: results from the International PNH Registry
Source: Ann Hematol. 2023 May 18;102(7):1637–44. doi: 10.1007/s00277-023-05269-4 (PMC10261189; doi:10.1007/s00277-023-05269-4)
Supplement: Supplementary file 6 — (DOCX 20 kb) [file 277_2023_5269_MOESM4_ESM.docx]

**Supplementary Table 2. eGFR at Last Follow-Up Stratified by Clone Size at Baseline^a^**

|  | **≤5%**  **(n=1006)** | **>5% to ≤10%**  **(n=221)** | **>10% to ≤30%**  **(n=443)** | **>30%**  **(n=1143)** |
| --- | --- | --- | --- | --- |
| n^b^ | 630 | 153 | 306 | 790 |
| eGFR, mL/min |  |  |  |  |
| Mean ± SD | 81.2±27.2 | 90.3±25.0 | 87.4±28.0 | 94.8±27.8 |
| n (%) |  |  |  |  |
| <30 | 13 (2.1) | 0 | 9 (2.9) | 16 (2.0) |
| ≥30 to <60 | 132 (21.0) | 18 (11.8) | 47 (15.4) | 87 (11.0) |
| ≥60 to <90 | 238 (37.8) | 57 (37.3) | 96 (31.4) | 187 (23.7) |
| ≥90 | 247 (39.2) | 78 (51.0) | 154 (50.3) | 500 (63.3) |

eGFR, estimated glomerular filtration rate; GPI, glycophosphatidylinositol; PNH, paroxysmal nocturnal hemoglobinuria.

^a^Baseline was defined as PNH onset (ie, disease start date) at the earliest reported GPI-deficient clone, date of PNH diagnosis, or PNH symptom.

^b^n indicates number of patients with nonmissing data.
