# Supplementary material for: Relationship of paroxysmal nocturnal hemoglobinuria (PNH) granulocyte clone size to disease burden and risk of major vascular events in untreated patients: results from the International PNH Registry
Source: Ann Hematol. 2023 May 18;102(7):1637–44. doi: 10.1007/s00277-023-05269-4 (PMC10261189; doi:10.1007/s00277-023-05269-4)
Supplement: Supplementary file 7 — (DOCX 20 kb) [file 277_2023_5269_MOESM5_ESM.docx]

**Supplementary Table 3. RBC Transfusion Rates from Baseline to Last Follow-Up Stratified by Clone Size at Baseline^a^**

|  | **≤5%**  **(n=1006)** | **>5% to ≤10%**  **(n=221)** | **>10% to ≤30%**  **(n=443)** | **>30%**  **(n=1143)** |
| --- | --- | --- | --- | --- |
| n^b^ | 992 | 219 | 434 | 1121 |
| RBC transfusions |  |  |  |  |
| No, n (%) | 511 (51.5) | 135 (61.6) | 259 (59.7) | 545 (48.6) |
| Yes, n (%) | 481 (48.5) | 84 (38.4) | 175 (40.3) | 576 (51.4) |
| RBC units transfused,^‡^ mean ± SD | 21.9±37.3 | 14.7±17.9 | 19.4±29.4 | 20.2±28.5 |
| Estimated rate of RBC transfusions (95% CI) | 1.8 (1.7–1.8) | 0.8 (0.7–0.8) | 1.0 (1.0–1.0) | 1.1 (1.0–1.1) |

GPI, glycophosphatidylinositol; PNH, paroxysmal nocturnal hemoglobinuria; RBC, red blood cell.

^a^Baseline was defined as PNH onset (ie, disease start date) at the earliest reported GPI-deficient clone, date of PNH diagnosis, or PNH symptom.

^b^n indicates total number of patients at risk.
